# Replay of incidentally encoded novel odors in the rat

**DOI:** 10.1007/s10071-024-01880-8

**Published:** 2024-06-14

**Authors:** Cassandra L. Sheridan, Lauren Bonner, Jonathon D. Crystal

**Affiliations:** Department of Psychological & Brain Sciences, Indiana University, Bloomington, IN USA

**Keywords:** Episodic memory, Replay, Incidental encoding, Unexpected question, Episodic-like memory, Olfaction

## Abstract

Although events are not always known to be important when they occur, people can remember details about such incidentally encoded information using episodic memory. Sheridan et al. (2024) argued that rats replayed episodic memories of incidentally encoded information in an unexpected assessment of memory. In one task, rats reported the third-last item in an explicitly encoded list of trial-unique odors. In a second task, rats foraged in a radial maze in the absence of odors. On a critical test, rats foraged in the maze, but scented lids covered the food. Next, memory of the third-last odor was assessed. The rats correctly answered the unexpected question. Because the odors used in the critical test were the same as those used during training, automatically encoding odors for the purpose of taking an upcoming test of memory (stimulus generalization) may have been encouraged. Here, we provided an opportunity for incidental encoding of novel odors. Previously trained rats foraged in the radial maze with entirely novel odors covering the food. Next, memory of the third-last odor was assessed. The rats correctly answered the unexpected question. High accuracy when confronted with novel odors provides evidence that the rats did not automatically encode odors for the purpose of taking an upcoming test, ruling out stimulus generalization. We conclude that rats encode multiple pieces of putatively unimportant information, and later replayed a stream of novel episodic memories when that information was needed to solve an unexpected problem.

**Supplementary Information:**

The online version contains supplementary material available at 10.1007/s10071-024-01880-8.

## Introduction

The capacity to recall a series of past events is a fundamental aspect of memory (Dede et al. 2016; Kurth-Nelson et al. 2016; Staresina et al. 2013). Often, humans can recollect past occurrences even when the information seemed trivial at the time of encounter (e.g., recalling yesterday’s breakfast). A crucial facet of such recollection is the absence of foreknowledge regarding the significance of the surrounding information for future memory retrieval. This form of memory retention involves incidental encoding, where information is not intentionally stored in memory, followed by an unexpected retrieval assessment (Crystal 2021; Zentall et al. 2001; Zhou et al. 2012).

Most methodologies for modeling episodic memory in non-human species involve some form of training. Through repeated exposure to related experiences, animals may recognize the significance of newly encountered information, prompting explicit encoding into memory. Furthermore, they might learn to anticipate the relevance of this information for future memory assessments. However, training animals to encode and report information explicitly in anticipated memory evaluations raises the possibility that non-episodic memory mechanisms could be employed to solve memory tasks in the absence of episodic memory. If animals can foresee the relevance of presented information to a forthcoming memory test during encoding, they might utilize non-episodic memory traces to pre-select planned actions. Consequently, during the memory test, animals could execute these planned actions without relying on the retrieval of episodic memories. This scenario poses a significant challenge to the notion that animals remember past events specifically by retrieving episodic memories (Crystal 2021; Zentall et al. 2001; Zhou et al. 2012). By contrast, when information is incidentally encoded and evaluated in an unforeseen memory test, it is not possible to transform this information into a planned future action. Planning future actions becomes difficult when the importance of information is unknown, and the nature of the unexpected memory test remains uncertain.

Recently, Sheridan et al. (2024) argued that rats replay episodic memories of incidentally encoded information in an unexpected assessment of memory. In one task, rats were trained to identify items that were the third-to-last item in a recently presented list (Sheridan et al. 2024). In this approach, rats were presented with a list of trial-unique odors and later were rewarded for selecting items that were previously encountered in a distinct ordinal position from the end of the list. Training took place in two distinct arenas featuring scented plastic lids covering food holes. Each list varied in length, ranging from 4 to 11 odors, presented within a contextual encoding environment, termed “list encoding”. Importantly, the unpredictability of list length prevented rats from anticipating its termination until being relocated to a different context. Following list presentation, rats underwent immediate memory assessments within a distinct memory assessment context, where they were presented with two odors from the list, termed “list memory assessment”. The correct choice corresponded to the third-to-last item in the list, while the incorrect choice was from a different randomly selected ordinal position in the list. Rats received rewards for selecting the third-to-last item. Given the training regimen, it is possible that this task involved explicit encoding of information in anticipation of answering an expected question.

In a second task, rats foraged for food in an eight-arm radial maze. Food was available at the end of each runway, covered by *unscented* lids. Initial foraging was restricted to four accessible baited arms (termed ‘‘study phase’’). Next, all eight arms were accessible (termed ‘‘test phase’’), and food was only available in arms that had not yet been visited. Accurate performance in the test phase was documented by visits to baited locations while avoiding the locations that were already depleted of food. When a rat forages in a study phase, it likely expects the continuation of foraging in the test phase. Moreover, it is well established that rats remember maze locations with respect to the global geometry of the room (Brown et al. 1993; Mazmanian and Roberts 1983; Olton and Collison 1979; Suzuki et al. 1980).

To arrange for incidental encoding, Sheridan et al. (2024) permitted rats to forage within the radial maze, but *scented* lids replaced the unscented lids on a single occasion. Next, instead of returning to the radial maze for a test phase, the rats were presented with a memory assessment for the odors encountered in the maze. Prior to this assessment, rats had never encountered scented lids while foraging in the maze, nor had they been subjected to an odor memory assessment after foraging. Consequently, based on earlier training, they could not know the significance of the maze odors or the possibility of being questioned about them in the future. Additionally, the scented lids in the maze were incidental to the rats’ foraging behavior (Timberlake and White 1990). Sheridan and colleagues found that when rats encountered odors while foraging on the radial maze and their memory for the sequence of encountered odors was evaluated after 0- and 15-minute delays, the rats successfully answered the unexpected question (Sheridan et al. 2024). They argued that these findings support the hypothesis that rats can replay a series of distinct events that were not known to be important when the events were encountered and subsequently remember this information when unexpectedly prompted to retrieve episodic memories.

However, there is a potential non-episodic memory solution available to the rats. According to stimulus generalization, it is possible that the rats treated odors in the critical test like they did in the earlier trained condition. Stimulus generalization is characterized by transfer (i.e., equivalent accuracy) and generalization decrement (i.e., lower accuracy), depending on the similarity of training and test conditions. The stimulus generalization hypothesis proposes that when the rats encountered a similar set of stimuli in training and testing conditions (e.g., lids, odors, displacement of lids, food), the rats may explicitly encode odors for the purpose of answering an upcoming memory test. By contrast, a generalization decrement hypothesis proposes that the rats rely on a broader set of stimulus conditions; broad stimulus conditions may include lid-odor-displacement-food and those that vary across conditions such as the pattern on the floor (black, white, etc.), spatial extent in the arena or maze (e.g., small, narrow, long, wide, etc.), curvature of the arena or maze (e.g., round, rectangular/straight), global geometric cues outside the arena or maze, prominent landmarks in the room (e.g., home cage, entrance to the room), etc. Stimulus generalization predicts equivalent performance in training and test conditions, whereas generalization decrement predicts that performance will be lower in test than in training conditions.

Sheridan et al. (2024) addressed the issue of stimulus generalization with a control condition that gave rats an opportunity to generalize to a novel encoding context. The rationale of the control condition is that if rats fail to generalize in the novel situation of the control condition, then it is unlikely that they generalized in the critical test described above. In the control condition, rats were presented with a sequence of odors in the familiar list-encoding context (list 1), followed by another sequence of odors in a new context (list 2). Next, the rats received a memory assessment where they had to choose between the third-last odor from list 1 and the third-last odor from list 2. It was proposed that if rats automatically encode odors in preparation for future tests (stimulus generalization), they would be expected to choose the odor from list 2, which was the literal third-last odor. Alternatively, if rats did not automatically encode odors for future tests (lack of stimulus generalization), they would likely select the odor from list 1, which was associated with the familiar list-encoding context. They found rats selected the odor from list 1, indicating a failure of generalization (Sheridan et al. 2024).

Although Sheridan et al. (2024) documented a failure of generalization in their control condition, the issue of stimulus generalization merits additional testing. Because the odors used in the critical test were the same as those used during training, it is possible that the automatic encoding of odors for the purpose taking an upcoming test of memory (stimulus generalization) was encouraged, which is a challenge to an interpretation based on replaying incidentally encoded episodic memories. It is possible that once the rats encountered these trained odors, they started to explicitly encode them into memory for the purpose of taking an upcoming test. Here, we provided rats with an opportunity for incidental encoding of *novel odors* to eliminate the possibility that familiarity with previously trained odors could contribute to successfully answering the unexpected question. Previously trained rats foraged in the radial maze with entirely novel odors covering the food. Next, memory of the third-last odor was assessed. If the similarity of training and testing odors contributed to passing the critical test described above, then the rats would be expected to fail to pass a critical test using novel odors (i.e., chance performance of 0.5). By contrast, if rats are capable of answering an unexpected question after incidental encoding, then they would be expected to perform at a high level of accuracy. High accuracy when confronted with novel odors would provide evidence that the rats did not automatically encode odors for the purpose of taking an upcoming test, ruling out stimulus generalization.

## Methods

### Subjects

Seven male Sprague-Dawley rats that previously served in Sheridan et al. (2024) were used (approximately 1.6 years old and approximately weighed 360 g at the start of the study). Rats were housed individually and maintained on a 12:12 light/dark cycle, with light onset at 7:30 a.m. and offset at 7:30 p.m. Water was available *ad libitum*, except during testing sessions. The rats received 45-mg chocolate and chow pellets (F0229 and F0164, respectively; Bio-Serv, Frenchtown, NJ) during experimental sessions. Daily rations were 15 g consisting of pellets consumed during experimental sessions and 5102-Rat-Diet (PMI Nutritional International, St. Louis, MO). All procedures followed the Guide for Care of Use of Laboratory Animals and were approved by the Bloomington Institutional Animal Care and Use Committee at Indiana University. Rats were previously trained on the following: *baseline list training* and *radial maze study-test task*, described below. Rats that were previously trained in Sheridan et al. (2024) were used here to minimize the number of animals, consistent with the 3 R’s principles, (Russell and Burch 1959), needed to conduct this study.

### Equipment

Two open-field arenas constructed from acrylic plexiglass served as distinctive contexts for list encoding and memory assessment. All arenas contained ‘‘food holes’’ that were used for the placement of scented lids. Each food hole was circular (5-cm diameter, 2.5-cm depth), which allowed a cup to be firmly snapped into place so that the cup lay flush with the floor and could be covered with a lid placed loosely on top. The arena used for list encoding was circular, with a 46-cm diameter floor, and a transparent 30-cm high wall. The floor pattern in the encoding arena consisted of 3 concentric circles, with the inner, middle, and outer circles that were black, white, and black, respectively. The inside of the arena consisted of 8 equidistant food holes positioned along the walls. The black arena that was used for the third to last memory assessment was square (61-cm length, 61-cm width, and 30-cm height) and had 12 equidistant food holes arranged along the perimeter of the walls. The radial maze (modified components from Lafayette Instruments) consisted of 8 arms (each 75-cm length) and a central hub (33-cm diameter). The arms and hub were surrounded with clear, plexiglass approximately 20-cm high and the arms were covered with a clear plexiglass lid that contained air holes. The ends of each arm contained a 5-cm food hole, inlayed in the floor, which allowed a cup to be firmly snapped into place so that the cup lay flush with the floor and could be covered with a lid placed loosely on top. Guillotine doors, made of clear plexiglass, separated the hub from each of the arms and could be individually raised using pneumatic cylinders via remote control. The open-field arenas and the radial maze were cleaned with 2% chlorhexidine solution after each animal completed its daily session.

### False-bottom cups

To ensure that the rats could not use the odor of the chocolate reward to select the correct lid, false-bottom cups were used in all memory assessments. Each memory assessment contained two cups: one for the correct item and another for the incorrect item. The metal grates (44.5-mm diameter, 179 holes, < 1 mm thick) were placed in the plastic cups at an angle above the pellet(s). The correct choice cup contained two chocolate pellets, separated by the thin metal grate (i.e., one chocolate pellet was placed below the metal grate, and the other pellet was placed above the grate). The incorrect choice cup contained two chocolate pellets placed below the metal grate, with no pellets placed above. The angle at which the metal grates were placed in the plastic cups resulted in the two chocolate pellets being approximately at the same level (i.e., height). The metal grates were fabricated to securely fit into the plastic cups, ensuring the rat could not remove the grate. At the end of each session, false-bottom cups were cleaned with 2% chlorhexidine solution. New false-bottom cups were prepared each day.

### Stimuli

Odors were presented with opaque plastic lids that were odorized by storing them in sealed plastic containers. Plastic containers were filled with 90 mL of an oil odorant or approximately 150 mL of a dry spice powder odorant, and lids were odorized for at least 2 weeks before being presented to the rats. A metal grating was used in each container to separate the lids and the odorants in order to prevent direct contact. Odorants were refreshed approximately every 2 months in order to maintain scent potency and consistency. Odorants used during *baseline list training* included: allspice, amaretto oil, anise, apple, apricot, banana, bay, black walnut, blackberry, blueberry oil, butterscotch oil, caraway seed, carob powder, celery seed, champagne, cheddar, cherry oil, chicory root, cilantro, cinnamon, clove, coconut, coffee oil, coriander, cumin, dill weed, fenugreek seed, garlic powder, hazelnut, hickory smoke, honey oil, horseradish, Indian curry, Irish cream oil, lavender, lemon zest, maple, marjoram, menthol-eucalyptus, Mexican oregano, mustard seed, nutmeg, onion powder, peach oil, pecan oil, pineapple oil, pistachio, pumpkin, raspberry, rosemary leaf, root beer, sage leaf, sesame oil, spearmint, spinach powder, strawberry oil, sumac, summer savory, sweet basil, tarragon, thyme, tomato, turmeric, wasabi, watermelon oil, white willow bark, Worcestershire. Odorants used during the *novel odor critical test* included: annatto, beet powder, black pepper, blue cheese, caramel, cardamom, castor oil, crème de menthe, English toffee, fennel seed, galangal, ginger, grape oil, hot chili, juniper berry, licorice oil, malt vinegar, mushroom, paprika, pina colada, soy sauce, tropical punch, tutti-frutti. All stimuli used as odors were purchased from The Great American Spice Company (Rockford, MI).

## General methods

One session was conducted each day, 5–7 days per week. During each session, the rat was removed from its home cage and placed in a holding cage, where it also resided during inter-trial intervals. Holding cages were the same as cages used in vivarium housing, except bedding, food, and water were not present. The behavioral testing room contained two to three open-field arenas, the 8-arm radial maze, and a platform for the holding cage. Arenas and the radial maze were elevated approximately 85 cm above the floor. During sessions, the default position for the experimenter was located in the middle of the two open-field arenas, approximately 1-m from the center of the arenas. For each trial, the experimenter removed the rat from the holding cage and placed the rat in the designated arena positioned with head pointed away from experimenter. Next, the experimenter returned to the default position where he/she remained with hands at his/her sides until the rat displaced the lid of the designated odor. The experimenter then removed the rat from the arena and returned it to the holding cage. When odors were presented multiple times during a session, new lids were used to prevent the rat from relying on scent marking. The following variables were randomized each session: the length of the lists, the odors used (sampled without replacement), and location of the odor lids. An individual rat advanced to subsequent phases of the procedure when it met a training criterion as outlined below, which allowed us to tailor advancement to the learning abilities of individual rats.

### Baseline list training

Rats were previously trained to identify items that were the third-to-last item in a recently presented list (Sheridan et al. 2024). Because learning the rules in our approach may be difficult for the rats, we used a training approach to optimize learning. Rats were tested in two distinctive arenas with “food holes” covered by scented opaque lids in training. To this end, we implemented training strategies that provided the rat with immediate feedback, allowed the rat to continue each trial until it displaced the lid to the correct item, and used a large reward for an initial correct choice. Since timely feedback promotes learning, we utilized a strategy that provided the rat with immediate feedback to facilitate learning of the third to last rule. To ensure that the rats could not track the odor of the chocolate reward, false-bottom cups were utilized during all memory assessments. Each memory assessment contained two false bottom cups: one for the correct item and another for the incorrect item. The incorrect item cup contained two chocolate pellets, both placed below a metal grate. The cup for the correct item contained two chocolate pellets, separated by a metal grate (i.e., one chocolate pellet was below the metal grate, and the other pellet was above the grate). Having one pellet above the grate provided immediate access to the reward after lid displacement, thereby minimizing the delay between the response and the delivery of the food reward. To further optimize learning, we provided the rat with feedback in every trial by allowing the rat to continue each trial if the initial response was incorrect. To this end, if the rat’s initial lid displacement was to an incorrect item, the trial continued until lid displacement of the correct item occurred (the second choice was not included in calculations of accuracy). Because our approach baited the cups of correct items and allowed the rat to continue in each trial following an initial incorrect choice, it is possible for the rat to choose randomly and still obtain many food rewards. Thus, to incentivize learning while implementing the features described above, we provided the rat with a large reward following a correct first choice. Specifically, a correct first response was rewarded with five additional chocolate pellets. To this end, immediately following the rat’s initial correct lid displacement, the experimenter delivered additional food pellets to the cup; the experimenter did not initiate delivery of the additional food reward until after the rat’s initial correct response had occurred. The large reward was not delivered if the initial choice was incorrect.

**Fig. 1:**
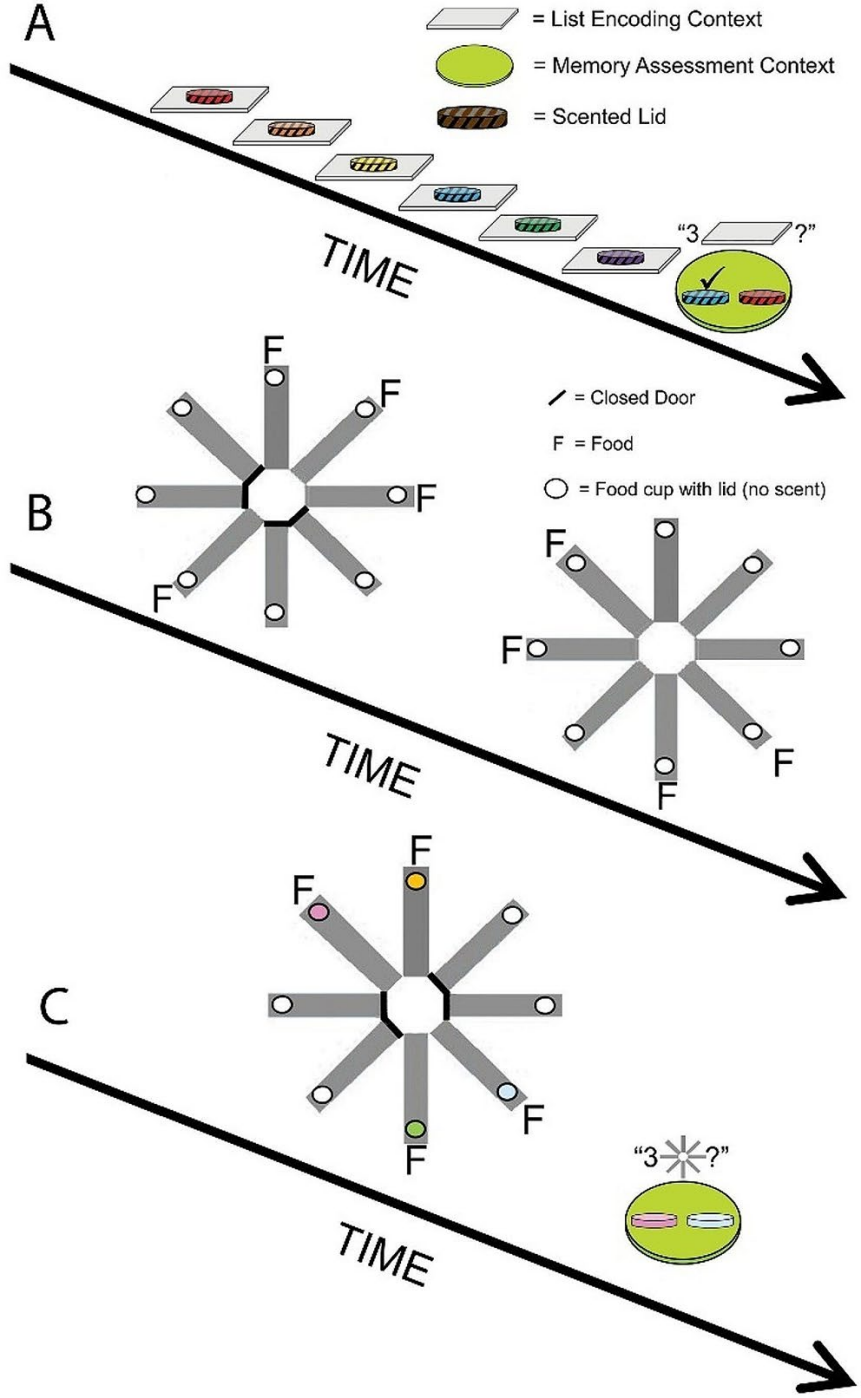
Replay of episodic memories after incidental encoding in an unexpected assessment of memory. (**A**) Rats are presented with a list of trial-unique odors and trained to pick items that occupied the third-last position from the end of the list. (**B**) Separately, rats forage for food (encountered below unscented lids on a radial maze). (**C**) On a critical test, rats forage on the radial maze but encounter food below completely novel scented lids. Next, rats are prompted to replay episodic memories to identify the third-last odor encoded during foraging. Scented lids in the radial maze provide a novel condition, never experienced in earlier training, and are incidental to foraging (incidental encoding). A list memory assessment after foraging is a novel situation (unexpected test)

Sessions consisted of approximately 4 trial-unique lists and corresponding memory assessments. Each list consisted of 4–11 trial-unique odors. Lists were presented to the rat in a distinctive encoding context, one item at a time; the number of items in the list was randomly selected for each trial (Fig. 1A). Each list item presentation consisted of a single cup and odorized lid placed at a randomly determined location and baited with a single chow pellet. The rat was removed from the holding cage and placed in the list encoding context facing away from the experimenter. A response was defined as the vertical or horizontal displacement of the lid from the cup. The rat remained in the encoding context until it displaced the lid and consumed the food reward. Immediately after the rat displaced the lid, it was removed from the arena and returned to the holding cage. This procedure continued until all items in the list were presented.

Immediately after completing each list presentation, the rat was moved to a distinctive context for the memory assessment (Fig. 1A). Memory assessments consisted of a choice between two odors presented in the list; one odor was from the third to last ordinal position in the list (the correct choice) and was baited with a single accessible chocolate pellet, whereas the other odor was not baited with an accessible pellet and was from another randomly selected ordinal position in the list (incorrect choice, foil odor). A correct choice was defined as the first lid displacement for the third to last list item, whereas an incorrect response was defined as the first lid displacement for an odor from a different ordinal position. Training continued for each rat until performance in the memory assessment met the following criterion: Mean accuracy observed in the third to last memory assessment was at least 75% in the last 6 consecutive sessions. Approximately 25 sessions were conducted in baseline list training.

### Radial maze study-test task

In study-test training (Fig. 1B), each food cup was baited with 1 chow pellet, which was covered by an unscented plastic lid. In the study (i.e., encoding) phase, 4 doors (randomly selected on each session for each rat) were opened. The rat was allowed to navigate the maze until all 4 pellets were consumed or 15 min elapsed, whichever occurred first. The rat was then removed, placed back into the holding cage, and the arms of the radial maze were cleaned. For the test (i.e., assessment) phase, all 8 doors were opened; at this stage, food was only available at the 4 arms not visited in the study phase. A visit to an arm was recorded if the rat placed all four paws in the arm, even if it did not displace the lid and obtain the food reward. The number of baited arms entered in the first four choices of the test phase (expressed as a proportion of four arms) was the dependent measure. This version of the radial maze procedure is a standard assessment of spatial working memory (Cowan 2016; Olton and Samuelson 1976; Roberts 1998). Rats advanced to the next phase when accuracy was at least 70% in the last four sessions. Approximately 14 sessions were conducted in radial maze study-test training.

### Alternation of baseline list training and radial-maze training

Baseline list training and study-test radial-maze training alternated across blocks of days. On any given day, only one task was scheduled to occur (list or radial maze, but not both). The number of consecutive days of list and radial maze was decreased across successive alternations. During this period before the critical test described below, the number of consecutive days of training was increased if the animal showed any signs of poor performance (e.g., below baseline criterion described above). Consequently, advancement to the critical test was preceded by a run of days (6 list, 2 radial maze, 4 list) with high accuracy in both list and radial maze tasks. The above precautions were used to ensure that high accuracy would be expected on the next day given the lack of any disruption in performance in the run up to the critical test. Approximately 39 sessions were conducted.

### Novel odor critical test

On a single occasion, the rat received a radial maze study phase with a randomly selected *novel-scented* lid placed on top of the food cup at the end of four accessible runways (Fig. 1C). In all other respects, the procedure was identical to a study phase in the radial maze. Next, the rat was immediately transferred to the memory assessment context. The odors in the memory assessment context consisted of the third to last odor from the radial maze (which was designated as the correct choice) and a foil randomly selected from the other odors presented in the radial maze (which was designated as the incorrect choice). The rat was rewarded for choosing the third to last item encountered in the radial maze. In the unexpected memory assessment of the critical test (Fig. 1C), the two odors were placed at equal distances (randomly selected from 6 of 12 food holes) away from the rat’s head to eliminate any bias in selecting an item nearest to the rat’s head.

## Results

**Fig. 2:**
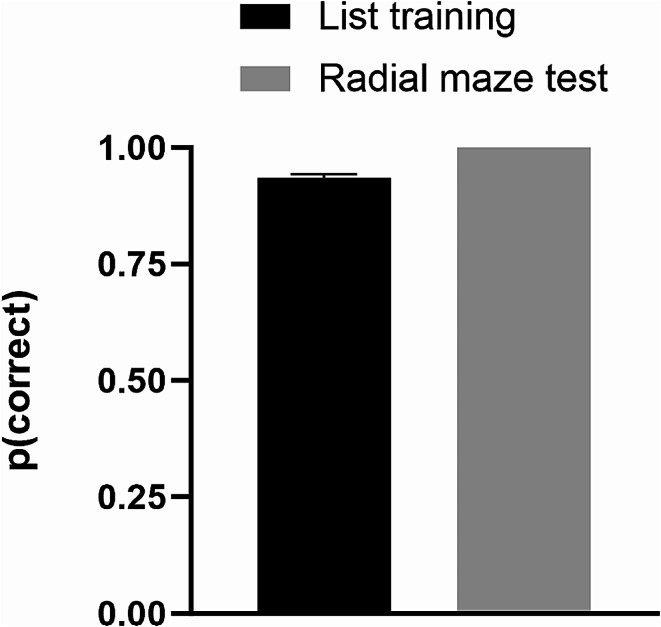
Rats replay incidentally encoded novel information using episodic memory. In list training (last six sessions of list training prior to the novel odor critical test, left bar), rats were asked to identify the ordinal position of items from a list of odors. In the novel odor critical test (on a single occasion, radial maze test, right bar), rats encountered entirely novel odors while foraging in the radial maze and their memory for the order of encountered novel odors was immediately assessed. Chance = 0.5. The error bar represents 1 SEM

When rats were asked to identify the ordinal position of odors from the list, rats selected the third to last odor at a high level of accuracy above chance (Fig. 2. List training, *t*(6) = 7.97, *p* < 0.001; independent measures t-test). When rats encountered *novel odors* while foraging on the radial maze and their memory for the order of encountered odors was immediately assessed, all rats correctly answered the unexpected question (Fig. 2. Novel odor critical test; binominal test, *n* = 7, *p* < 0.01). Notably, the data for the novel odor critical test in Fig. 2 come from a single test conducted with each rat (see Online Supplemental Resource, Table S1); thus, the data were obtained before the opportunity for new learning.

## Discussion

We provided rats with an opportunity to incidentally encode completely novel odors followed by an unexpected assessment of memory. This approach eliminates the possibility that familiarity with previously trained odors could contribute to successfully answering the unexpected question. If the rats were using episodic memory to replay incidentally encoded novel odors, then they should correctly solve the memory assessment at high levels of accuracy. In contrast, if the rats were using stimulus generalization to solve the novel odor critical test, then their performance in the critical test would be at the level expected by chance. We found that when memory of the third-last odor was assessed, all rats correctly answered the unexpected question. High accuracy when confronted with novel odors provides further evidence that the rats did not automatically encode odors for the purpose of taking an upcoming test, ruling out stimulus generalization.

Here we wanted to address the possibility of stimulus generalization in rats’ successful performance in a critical test of incidental encoding followed by an unexpected assessment of memory. Although Sheridan et al. (2024) conducted a control condition (described above) to address the issue of stimulus generalization and found a failure of generalization, the issue of stimulus generalization is a major concern; therefore, multiple lines of evidence would strengthen any conclusion on this issue. In our previous work, the same odors that were used during baseline list training (randomly sampled from a large pool of odors) were also used during the critical test and the control condition. Although it is unlikely that a specific sequence of odors in the critical test or control condition were presented previously to the rats in baseline list training, the familiarity of specific odors could potentially increase the likelihood that they generalize from training to testing conditions. Therefore, it is possible that once the rats encountered familiar odors during the critical test, they automatically began to explicitly encode the odors into memory for the purpose of taking a future test of memory. The possibility just described is a major threat to the episodic memory central hypothesis. This concern is precluded by the current study through the use of completely novel odors which eliminates the possibility that previously trained odors triggered stimulus generalization. A number of other cues may promote generalization such as lids, odors, displacement of lids, and food. However, our earlier control condition provided a strong test for the ability of such cues to promote generalization, and in that case, we documented a failure of generalization.

Although the rats used in this study had experience in a previous experiment (Sheridan et al. 2024), it is unlikely that the earlier test experience influenced the current data. In Sheridan et al., the rats experienced a single critical test with a 0-minute delay and a single critical test with a 15-minute delay. The average lag between the critical tests in Sheridan et al. and the novel odor test reported here was approximately 8.4 months. Moreover, two tests are unlikely to produce substantial learning, especially given their remoteness, and given that our overall experience with rats’ performance in the basic replay task is characterized by relatively modest and slow learning.

To address the possibility that rats used food odors to guide choices, we used a classic approach to equate food odors in both the correct and incorrect choices. Both cups were baited with two pellets, with one accessible in the correct cup and none accessible in the incorrect cup. Notably, we ensured that the distance between the pellets and the rat’s nose at the time of choice was equal for all pellets, and numerous holes permitted air exchange across the false bottom cups (see methods for details). Therefore, it is unlikely that the rats could use food odors to guide choices.

The proposal that animals represent episodic memories has historically been viewed with some controversy (Corballis 2013; Crystal and Suddendorf 2019). Although several lines of evidence now converge on the conclusion that rats are a suitable model for episodic memory (Crystal 2021, 2024; in press), the history of controversy on this topic means that carefully designed experimental approaches are needed to make the case in a compelling fashion. Moreover, the notion that rats replay episodic memories bears special scrutiny because this line of work proposes an especially complex process. Namely, this approach argues that rats represent multiple items in episodic memory, they represent the order of such items, and they are capable of searching the representational space in episodic memory to find a target that occupies a specific ordinal position within a recently presented list of items. Therefore, we have conducted a number of tests to rule out the use of familiarity and working memory in our previous work (Panoz-Brown et al. 2018); reviewed in (Crystal 2021). Here we advanced this effort by using an approach that is devoid of stimulus generalization. Our conclusion is that rats can replay a stream of multiple episodic memories of items that were not known to be important at the time of encoding (incidental encoding) and were not known to be the subject of an upcoming test of memory (unexpected question).

In animal models of episodic memory, the central hypothesis is that at the moment of the memory assessment, an animal remembers back in time to a specific earlier event or episode to correctly solve the task. However, most animal models of episodic memory involve some form of training. In contrast, if rats were automatically encoding odors for the purpose of taking an upcoming test of memory (i.e., stimulus generalization), the rats may engage in explicit encoding and utilize non-episodic memory traces to pre-select planned actions, without remembering back in time to an earlier event or episode. These results are consistent with the hypothesis that rats can replay a stream of unique events that were not known to be important when the events were encountered and report this information when unexpectedly asked to search episodic memories. In addition, these results further validate our animal model of episodic memory, so that it can be used to study diseases of human memory, such as Alzheimer’s disease.

Validating an animal model of episodic memory requires ruling out non-episodic memory solutions to the memory problem. Accordingly, these experiments generally require the animal to learn complex or abstract rules (e.g., third last item in a list of unpredictable length). Our strategy for developing animal models of episodic memory is to focus on the aspects of the animal’s behaviors that it relies on in its ecological niche (Shettleworth 2009). In a series of experiments (Bratch et al. 2016; Panoz-Brown et al. 2016, 2017, 2018; Sheridan et al. 2024), we leverage the well-known proficiency of rodent olfaction (April et al. 2013). In another series of experiments (Babb and Crystal 2005, 2006a, b; Crystal and Alford 2014; Crystal et al. 2013a; Crystal, Ketzenberger, et al., 2013b; Crystal and Smith 2014; Zhou and Crystal 2009, 2011; Zhou et al. 2012), we leverage the well-known proficiency of rodent spatial navigation (Olton and Samuelson 1976). Utilizing these adaptive specializations in olfaction and spatial navigation allow us to successfully layer on top of these key capacities complex or abstract contingencies. We suspect that such layering would not be successful if we did not focus on capacities that are well-developed in rats (e.g., using visual stimuli). In contrast, when studying episodic memory in humans, odor is not typically used as a stimulus. Notably, visual acuity is more pronounced in people than is olfaction. Accordingly, visual stimuli are widely used in human episodic memory. Nonetheless, we argue that experiments in rats using olfaction or spatial navigation may tap into underlying mechanisms of episodic memory that may be used to develop animal models of human cognition.

## Electronic supplementary material

Below is the link to the electronic supplementary material.

Supplementary Material 1

## Data Availability

Data from each rat are provided in supplemental information (Table S1).

## References

[CR1] April LB, Bruce K, Galizio M (2013) The magic number 70 (plus or minus 20): variables determining performance in the rodent odor span task. Learn Motiv 44(3):143–158 23729864 10.1016/j.lmot.2013.03.001 PMC3665427

[CR2] Babb SJ, Crystal JD (2005) Discrimination of what, when, and where: implications for episodic-like memory in rats. Learn Motivation 36:177–189. 10.1016/j.lmot.2005.02.009

[CR3] Babb SJ, Crystal JD (2006a) Discrimination of what, when, and where is not based on time of day. Learn Behav 34:124–130. 10.3758/bf03193188 16933798 10.3758/bf03193188

[CR4] Babb SJ, Crystal JD (2006b) Episodic-like memory in the rat. Curr Biol 16:1317–1321. 10.1016/j.cub.2006.05.025 16824919 10.1016/j.cub.2006.05.025

[CR5] Bratch A, Kann S, Cain JA, Wu J-E, Rivera-Reyes N, Dalecki S, Arman D, Dunn A, Cooper S, Corbin HE, Doyle, AR, Pizzo, MJ, Smith, AE, Crystal, JD (2016) Working memory systems in the rat. Curr Biol 26(3):351–355. 10.1016/j.cub.2015.11.068 26776732 10.1016/j.cub.2015.11.068 PMC4747793

[CR6] Brown MF, Rish PA, VonCulin JE, Edberg JA (1993) Spatial guidance of choice behavior in the radial-arm maze. J Exp Psychol Anim Behav Process 19(3):195 8340766

[CR7] Corballis MC (2013) Mental time travel: a case for evolutionary continuity. Trends Cogn Sci 17(1):5–6. http://linkinghub.elsevier.com/retrieve/pii/S1364661312002458 23153675 10.1016/j.tics.2012.10.009

[CR8] Cowan N (2016) The many faces of working memory and short-term storage [journal article]. Psychon Bull Rev 1–13. 10.3758/s13423-016-1191-6 10.3758/s13423-016-1191-6 27896630

[CR9] Crystal JD (2021) Evaluating evidence from animal models of episodic memory. J Experimental Psychology: Anim Learn Cognition 47(3):337 10.1037/xan0000294 34618532

[CR10] Crystal JD (2024) Temporal foundations of episodic memory. Learn Behav 52:35–50. 10.3758/s13420-023-00608-x 37932642 10.3758/s13420-023-00608-x

[CR11] Crystal JD (in press) Mental time travel in the rat. Philisophical Trans Royal Soc B. 10.1098/rstb.2023.0404 10.1098/rstb.2023.0404 PMC11449164 39278253

[CR12] Crystal JD, Alford WT (2014) Validation of a rodent model of source memory. Biol Lett 10(3):20140064. 10.1098/rsbl.2014.0064 24647728 10.1098/rsbl.2014.0064 PMC3982441

[CR15] Crystal JD, Smith AE (2014) Binding of episodic memories in the rat. Curr Biol 24(24):2957–2961. 10.1016/j.cub.2014.10.074 25466681 10.1016/j.cub.2014.10.074 PMC4269559

[CR16] Crystal JD, Suddendorf T (2019) Episodic memory in nonhuman animals? Curr Biol 29(24):R1291–R1295 31846670 10.1016/j.cub.2019.10.045

[CR14] Crystal JD, Ketzenberger JA, Alford WT (2013b) Practicing memory retrieval improves long-term retention in rats. Curr Biol 23(17):R708–709. 10.1016/j.cub.2013.07.044 24028945 10.1016/j.cub.2013.07.044 PMC4005794

[CR13] Crystal JD, Alford WT, Zhou W, Hohmann AG (2013a) Source memory in the rat. Curr Biol 23(5):387–391. 10.1016/j.cub.2013.01.023 23394830 10.1016/j.cub.2013.01.023 PMC3595394

[CR17] Dede AJO, Frascino JC, Wixted JT, Squire LR (2016) Learning and remembering real-world events after medial temporal lobe damage. Proc Natl Acad Sci 113(47):13480–13485. 10.1073/pnas.1617025113 27821761 10.1073/pnas.1617025113 PMC5127365

[CR18] Kurth-Nelson Z, Economides M, Dolan, Raymond J, Dayan P (2016) Fast sequences of non-spatial state representations in humans. Neuron 91(1):194–204. 10.1016/j.neuron.2016.05.028 27321922 10.1016/j.neuron.2016.05.028 PMC4942698

[CR19] Mazmanian DS, Roberts WA (1983) Spatial memory in rats under restricted viewing conditions. Learn Motiv 14(2):123–139

[CR20] Olton DS, Collison C (1979) Intramaze cues and odor trails fail to direct choice behavior on an elevated maze. Anim Learn Behav 7(2):221–223

[CR21] Olton DS, Samuelson RJ (1976) Remembrance of places passed: spatial memory in rats. J Exp Psychol Anim Behav Process 2(2):97–116

[CR23] Panoz-Brown D, Corbin HE, Dalecki SJ, Gentry M, Brotheridge S, Sluka CM, Wu J-E, Crystal JD (2016) Rats remember items in context using episodic memory. Curr Biol 26(20):2821–2826 27693137 10.1016/j.cub.2016.08.023 PMC5117826

[CR22] Panoz-Brown D, Carey LM, Smith AE, Gentry M, Sluka CM, Corbin HE, Wu J-E, Hohmann AG, Crystal JD (2017) The chemotherapeutic agent paclitaxel selectively impairs reversal learning while sparing prior learning, new learning and episodic memory. Neurobiol Learn Mem 144:259–270. 10.1016/j.nlm.2017.08.001 28811227 10.1016/j.nlm.2017.08.001 PMC5621653

[CR24] Panoz-Brown D, Iyer V, Carey LM, Sluka CM, Rajic G, Kestenman J, Gentry M, Brotheridge S, Somekh I, Corbin HE, Tucker KG, Almeida B, Hex SB, Garcia KD, Hohmann AG, Crystal JD (2018) Replay of episodic memories in the rat. Curr Biol 28(10):1628–1634e1627. 10.1016/j.cub.2018.04.006 29754898 10.1016/j.cub.2018.04.006 PMC5964044

[CR25] Roberts WA (1998) Principles of animal cognition. McGraw-Hill

[CR26] Russell WMS, Burch RL (1959) The principles of humane experimental technique. Methuen

[CR27] Sheridan CL, Lang S, Knappenberger M, Albers C, Loper R, Tillett B, Sanchez J, Wilcox A, Harrison T, Panoz-Brown D, Crystal JD (2024) Replay of incidentally encoded episodic memories in the rat. Curr Biol 34(3):641–647e645. 10.1016/j.cub.2023.12.043 38218186 10.1016/j.cub.2023.12.043

[CR28] Shettleworth SJ (2009) Cognition, evolution, and behavior. Oxford University Press

[CR29] Staresina BP, Alink A, Kriegeskorte N, Henson RN (2013) Awake reactivation predicts memory in humans. Proceedings of the National Academy of Sciences, 110(52), 21159–21164. 10.1073/pnas.1311989110 10.1073/pnas.1311989110 PMC3876238 24324174

[CR30] Suzuki S, Augerinos G, Black AH (1980) Stimulus control of spatial behavior on the eight-arm maze in rats. Learn Motiv 11(1):1–18

[CR31] Timberlake W, White W (1990) Winning isn’t everything: rats need only food deprivation and not food reward to efficiently traverse a radial arm maze. Learn Motiv 21(2):153–163

[CR32] Zentall TR, Clement TS, Bhatt RS, Allen J (2001) Episodic-like memory in pigeons. Psychon Bull Rev 8:685–690 11848586 10.3758/bf03196204

[CR33] Zhou W, Crystal JD (2009) Evidence for remembering when events occurred in a rodent model of episodic memory. Proc Natl Acad Sci USA 106(23):9525–9529. 10.1073/pnas.0904360106 19458264 10.1073/pnas.0904360106 PMC2695044

[CR34] Zhou W, Crystal JD (2011) Validation of a rodent model of episodic memory. Anim Cogn 14(3):325–340. 10.1007/s10071-010-0367-0 21165663 10.1007/s10071-010-0367-0 PMC3079053

[CR35] Zhou W, Hohmann AG, Crystal JD (2012) Rats answer an unexpected question after incidental encoding. Curr Biol 22(12):1149–1153 22633809 10.1016/j.cub.2012.04.040 PMC3376895

