# Correction: Replay of incidentally encoded novel odors in the rat

**DOI:** 10.1007/s10071-025-01993-8

**Published:** 2025-07-26

**Authors:** Cassandra L. Sheridan, Lauren Bonner, Jonathon D. Crystal

**Affiliations:** Department of Psychological & Brain Sciences, Indiana University, Bloomington, IN USA

**Correction: Animal Cognition (2024) 27:43**

10.1007/s10071-024-01880-8

In this article the ‘Conflict of Interest’ statement was missing and should have been “Jonathon D. Crystal is an editor for the journal and has not taken part in the manuscript’s peer review process.” The original article has been corrected.

