# Supplementary material for: Replay of incidentally encoded novel odors in the rat
Source: Anim Cogn. 2024 Jun 14;27(1):43. doi: 10.1007/s10071-024-01880-8 (PMC11178560; doi:10.1007/s10071-024-01880-8)
Supplement: Supplementary file 1 — Supplementary Material 1 [file 10071_2024_1880_MOESM1_ESM.docx]

**Supplemental Information**

| **Subject** | **p(correct)** | |
| --- | --- | --- |
|  | **Baseline list training** | **Novel odor critical test** |
| NZ-04 | 0.93 | 1 |
| NZ-06 | 0.92 | 1 |
| NZ-08 | 0.91 | 1 |
| NZ-10 | 0.96 | 1 |
| NZ-12 | 0.95 | 1 |
| NZ-14 | 0.96 | 1 |
| NZ-15 | 0.91 | 1 |
| **Mean ± SEM** | **0.93 ± 0.01** | **1 ± 0** |

**Table S1. Performance of individual rats in the novel odor critical test, corresponding to data presented in Figure 2.**

The expected level of accuracy (chance) is 0.5.
